# Supplementary material for: Developmental and Molecular Effects of C-Type Natriuretic Peptide Supplementation in In Vitro Culture of Bovine Embryos
Source: Int J Mol Sci. 2024 Oct 11;25(20):10938. doi: 10.3390/ijms252010938 (PMC11507445; doi:10.3390/ijms252010938)
Supplement: Supplementary file 1 [file ijms-25-10938-s001.zip › ijms-3211287-supplementary.pdf]

Supplementary Material

Table S1: Gene symbol, functions and primers assay ID used for microfluidic expression analyses (Biomark HD System - Fluidigm).

Gene Symbol	Function	Assay ID*
**GAPDH**	Reference gene	Bt03210912_g1
**ACTB**	Reference gene	PA5-16914
**CD40**	Apoptosis	Bt03817804_g1
**BID**	Apoptosis	Bt03241255_m1
**BAX**	Apoptosis	Bt03211777_g1
**CASP3**	Apoptosis	Bt03250954_g1
**NPPA**	Cell development	Bt03223175_g1
**NPPB**	Cell development	Bt04301375_g1
**NPR1**	Cell development	Bt04297034_g1
**IMPDH1**	Cell development	Bt00995384_m1
**NLRP5**	Cell development	Bt03218031_m1
**BMP15**	Cell development	Bt03286494_u1
**NANOG**	Cell development	Bt03220541_m1
**SOX2**	Cell development	Bt03278318_s1
**NRP1**	Cell development	Bt04284287_m1
**NRP2**	Cell development	Bt04284287_m1
**BDNF**	Cell development	Bt04316732_m1
**FSHR**	Cell development	Bt03212674_m1
**IGFBP4**	Cell development	Bt03259500_m1
**ADCY6**	Cell development	Bt03816767_m1
**ADCY9**	Cell development	Bt04287024_m1
**EGFR**	Cell development	AJT96D7
**HSPA1A**	Cell development	Bt03292670_g1
**NPPC**	Cell development	Bt03212844_m1
**NPR2**	Cell development	Bt04316732_m1
**POU5F1**	Cell development	Bt03223846_g1
**ELF5**	Cell development	Bt03220307_m1
**HSPA5**	Oxidative stress	Bt03244880_m1
**GFPT2**	Oxidative stress	Bt03250351_m1
**GLRX2**	Oxidative stress	Bt03229700_m1
**SOD1**	Oxidative stress	Bt03215423_g1
**GPX1**	Oxidative stress	Bt03259217_g1
**FDX1**	Oxidative stress	Bt03217449_m1
**CAT**	Oxidative stress	Bt03228713_m1
**FADS2**	Lipid metabolism	Bt03256255_g1
**SCD**	Lipid metabolism	Bt04307476_m1
**ACSL1**	Lipid metabolism	Bt03248469_m1

**ACAT1**	Lipid metabolism	Bt03238649_g1
**PNPLA2**	Lipid metabolism	Bt03234129_g1
**PLIN2**	Lipid metabolism	Bt03212182_m1
**PLIN3**	Lipid metabolism	Bt03230537_m1
**AGPAT9**	Lipid metabolism	Bt04292093_m1
**AGPAT1**	Lipid metabolism	Bt03224587_g1
**SREBF1**	Lipid metabolism	Bt03276370_m1
**ACACA**	Lipid metabolism	Bt03213389_m1
**ACSL3**	Lipid metabolism	Bt04282138_m1
**ACSL6**	Lipid metabolism	Bt03231692_m1
**ELOVL1**	Lipid metabolism	Bt03286627_s1
**ELOVL2**	Lipid metabolism	Bt03256849_m1
**ELOVL4**	Lipid metabolism	Bt03270721_m1
**ELOVL5**	Lipid metabolism	Bt03235956_m1
**ELOVL6**	Lipid metabolism	Bt00907566_m1

* ThermoFischer Scientific

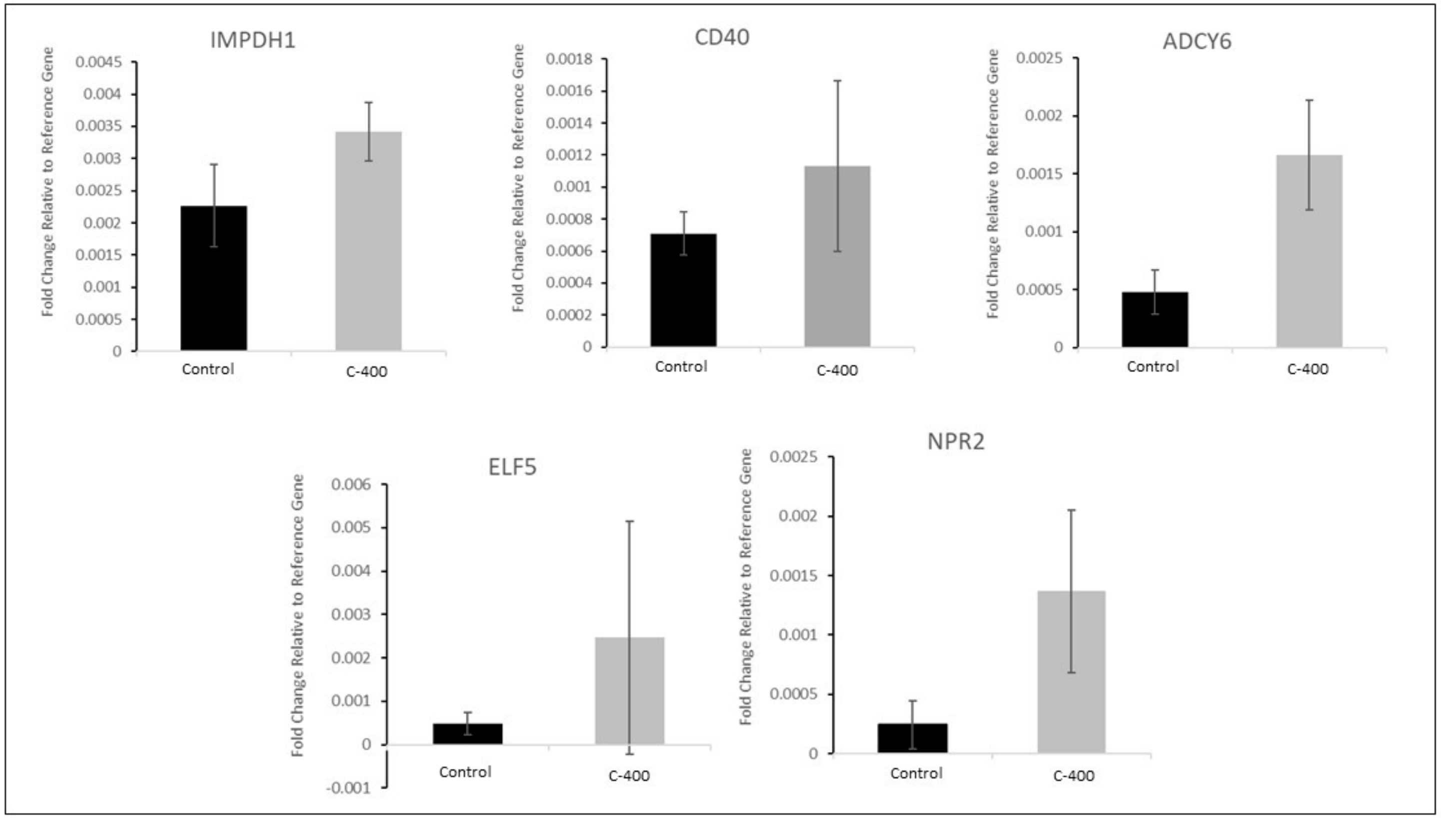

Figure S1- Upregulated transcription observed in bovine morulas after treatment with CNP. The relative abundance of transcripts was selected by the Fold change analysis (with magnitude greater than 1.5 times, that is, with the threshold > 1.5). Control (no treatment) and C-400 (400 nM of CNP).

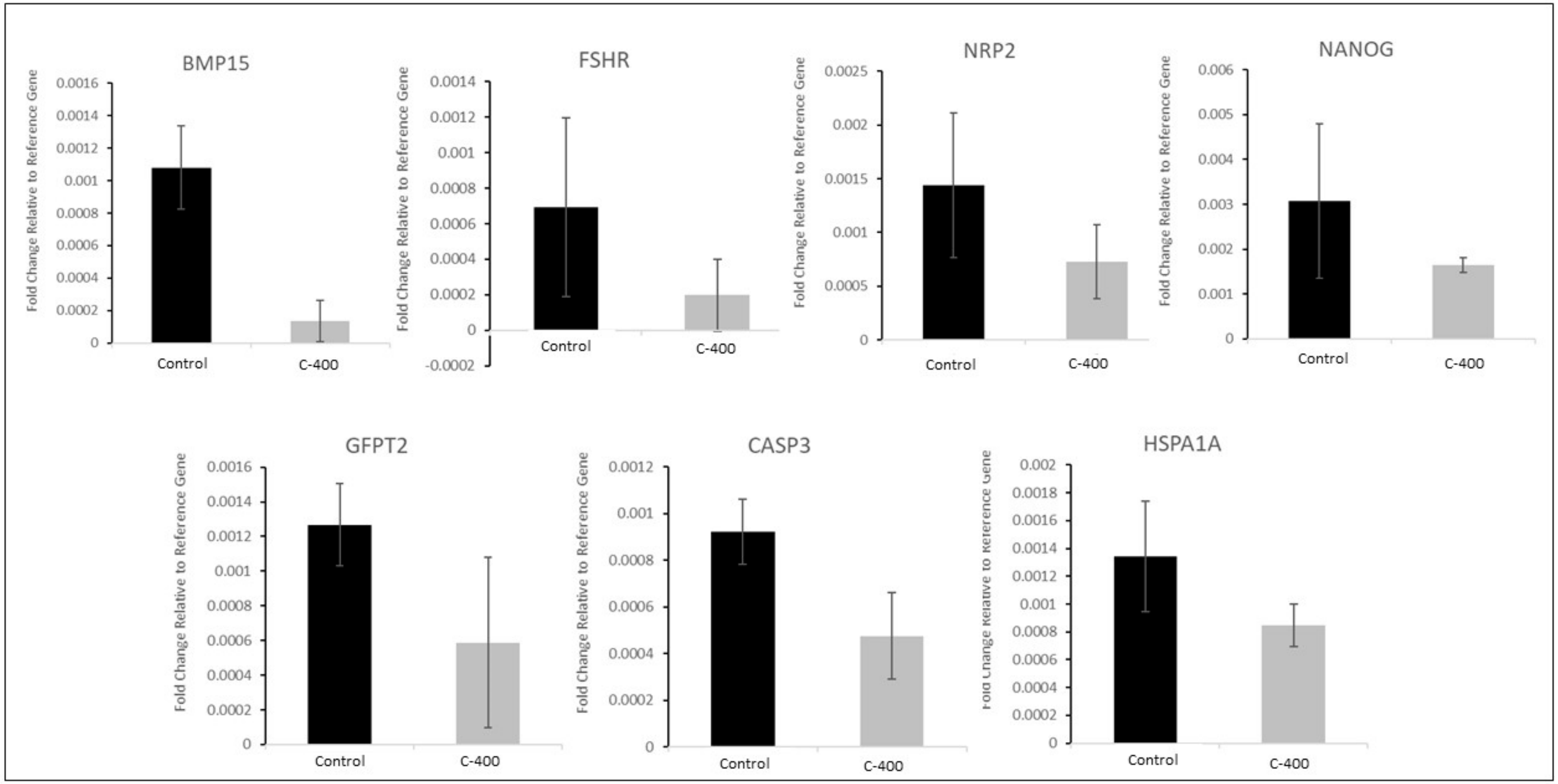

Figure S2- Downregulated transcription observed in bovine morulas after treatment with CNP. The relative abundance of transcripts was selected by the Fold change analysis (with magnitude greater than 1.5 times, that is, with the threshold > 1.5). Control (no treatment) and C-400 (400 nM of CNP).

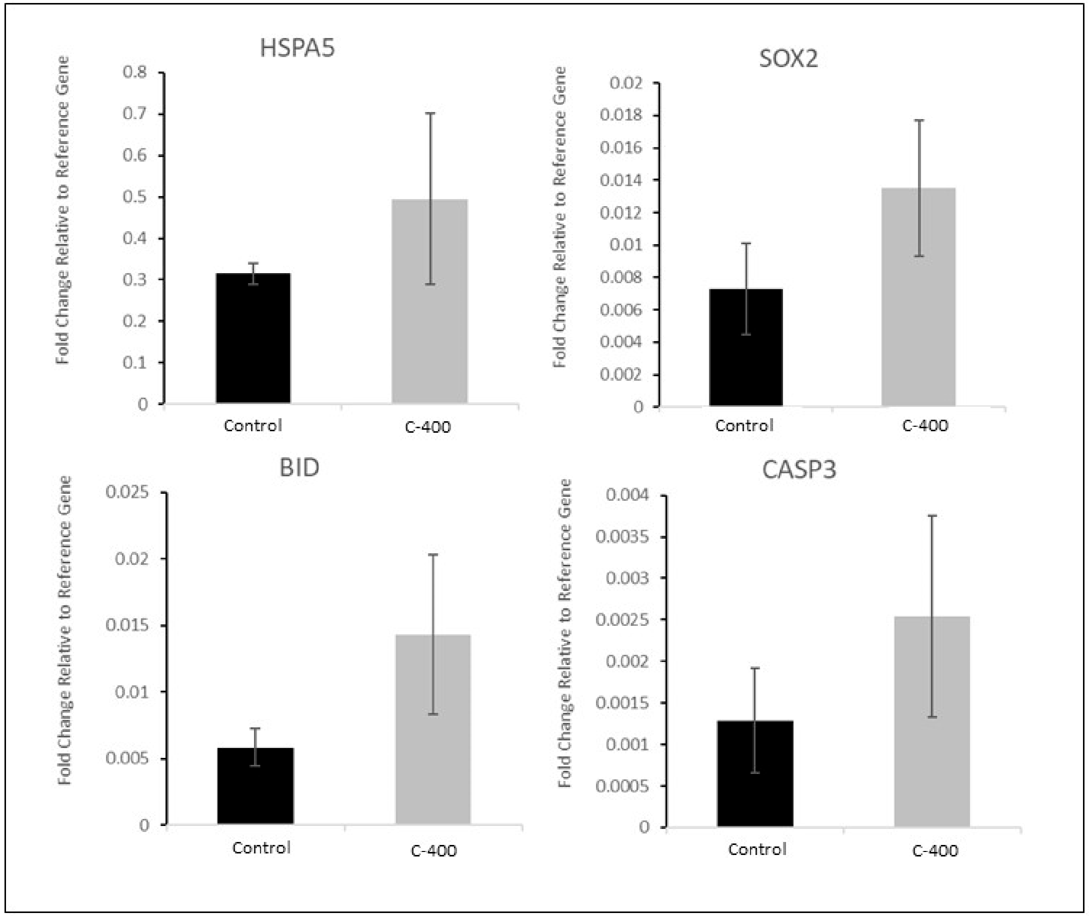

Figure S3- Upregulated transcription observed in bovine blastocytes after inclusion of CNP in *in vitro* culture. The relative abundance of transcripts was selected by the Fold change analysis (with magnitude greater than 1.5 times, that is, with the threshold > 1.5). Control (no treatment) and C-400 (400 nM of CNP).

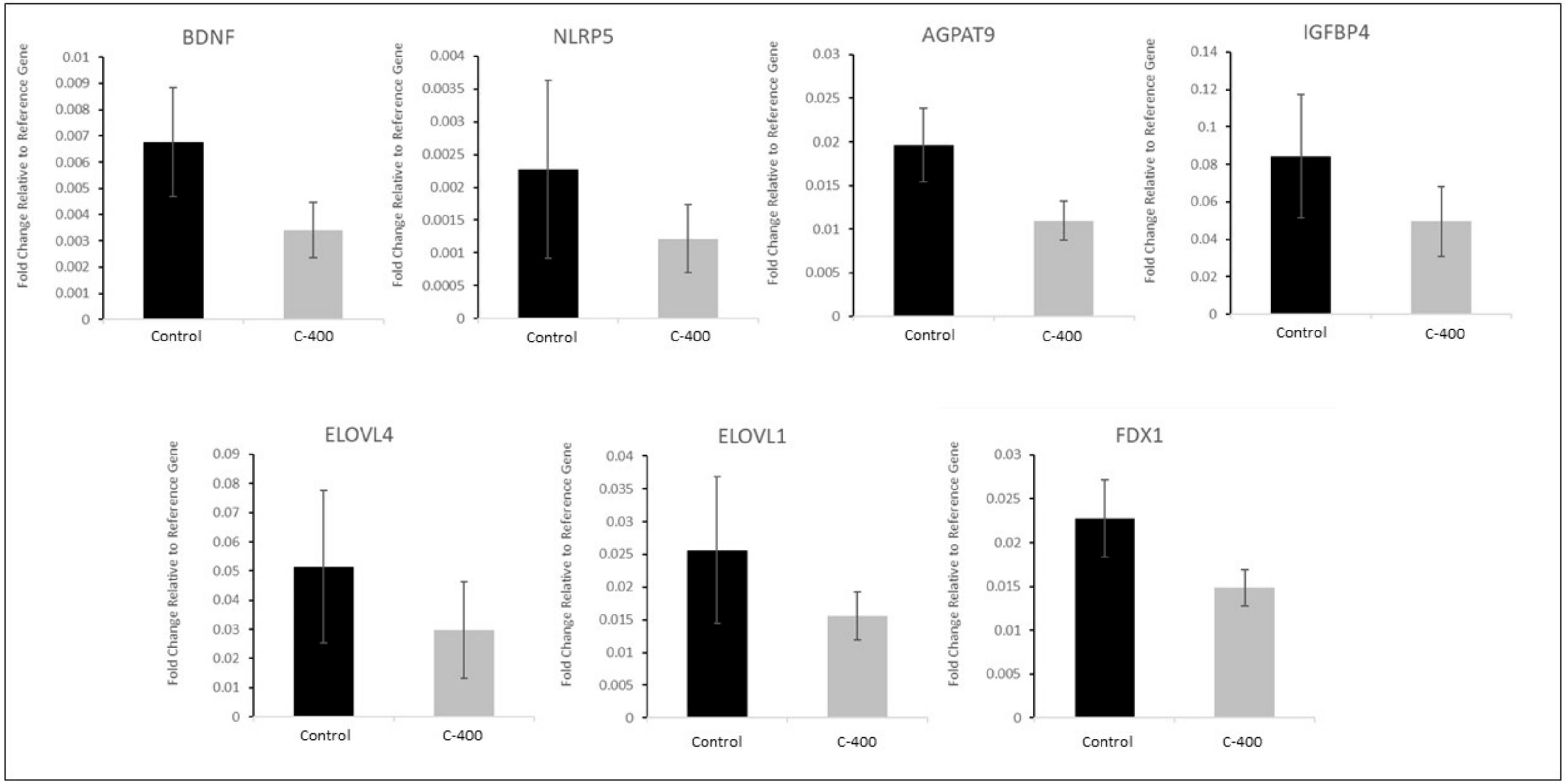

Figure S4- Downregulated transcription observed in bovine blastocytes after inclusion of CNP in *in vitro* culture. The relative abundance of transcripts was selected by the Fold change analysis (with magnitude greater than 1.5 times, that is, with the threshold > 1.5). Control (no treatment) and C-400 (400 nM of CNP).

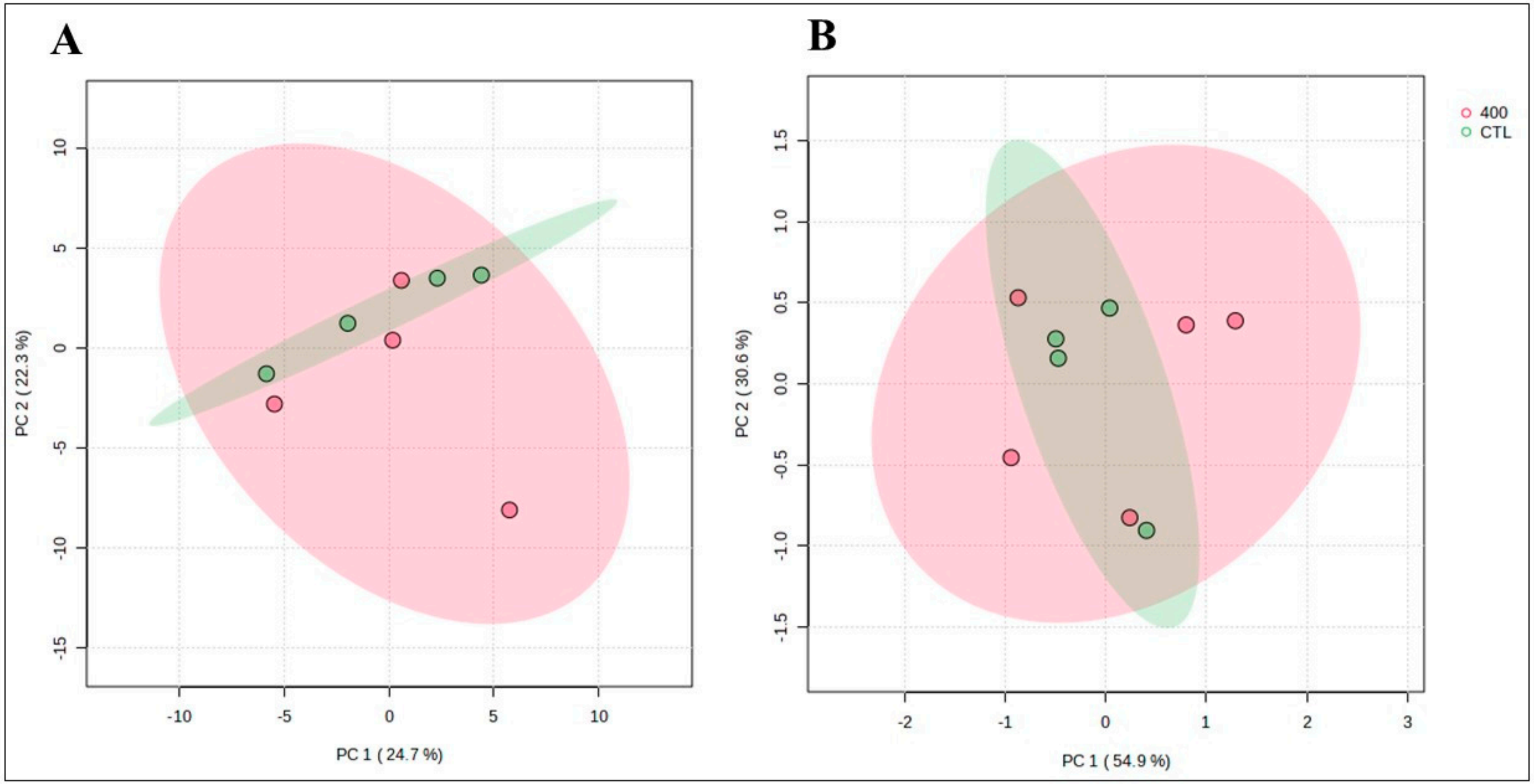

Figure S5- Multivariate analysis plots of the abundance of transcripts derived from untreated (control) and CNP-treated morula. **(A)** PCA plot of morula from control and treated groups; **(B)** PCA plot of blastocyst treated with CNP and control group (n = 4 / group). Control (no treatment) and C-400 (400 nM of CNP).
